# Immunological characteristics of IgG4-related Kuttner tumors

**DOI:** 10.1097/MD.0000000000030175

**Published:** 2022-09-02

**Authors:** Su Geun Kim, Chan Mi Lee, Yong Tae Hong

**Affiliations:** a Department of Otolaryngology-HNS, Research Institute for Clinical Medicine of Jeonbuk National University, Biomedical Research Institute of Jeonbuk National University Hospital, Jeonbuk, Korea.

**Keywords:** IgG4, immunoglobulin, Kuttner tumor, submandibular gland

## Abstract

Immunoglobulin G4-related disease (IgG4-RD) has recently been well recognized and Kuttner tumor is known to be a chronic sclerosing sialadenitis, representing the focal manifestation of IgG4-RD, in the submandibular gland (SMG). This study is to evaluate the immunologic features of IgG4-related Kuttner tumor in the SMG. We retrospectively chose 13 patients who were confirmed as having Kuttner tumor by surgical biopsy between May 2012 and January 2019. The fine-needle aspiration cytology, serum antibody levels (anti-Ro antibodies, anti-La antibodies), IgG serum levels (total IgG and IgG4), and immunohistochemical findings for IgG and IgG4-positive plasma cells were reviewed. The cytologic results found that 7 of the 9 cases were reported as chronic sialoadenitis, and the other 3 as benign lymphoproliferative lesion. The serum levels of autoantibodies, Sjögren-syndrome-related antigen A/Ro-Ab and Sjögren-syndrome-related antigen A/Ro-La, showed all normal values of serum level. The serum level of IgG was increased in only 4 among the cases. However, the IgG4 levels were significantly increased in 11 among the cases. In all the patients who received resection of SMG, immunohistochemical findings showed all positive for IgG4-RD, with elevated numbers of IgG and IgG4-positive plasma cells. The evaluation of IgG4 serum level should be very informative for the diagnosis of this tumor before surgery. Fine-needle aspiration cytology with ultrasound guidance are not conclusive in this study. The immunological study including IgG4 serum level should be required for proper diagnosis and treatment, with clinical features of the Kuttner tumor. The level of evidence was IV.

## 1. Introduction

Kuttner tumor is a benign inflammatory condition of the salivary gland predominantly involving the submandibular gland (SMG). This tumor may occur in other major and minor salivary glands, unilaterally or bilaterally, including the sublingual and parotid glands.^[1]^ This tumor should be differentiated from other benign or malignant tumors in the SMG. The salivary gland tumors show a great deal of morphological diversity and mimic the clinical appearance of malignancy in the salivary gland.^[2]^ The pathogenesis of Kuttner tumor in the SMG is not very well understood. Three factors have been mainly suggested: sialolith, inflammation of the salivary duct, and autoimmune reactions. Sialolithiasis by accumulation of calcium salts had been proposed as the most common cause for Kuttner tumor of the SMG.^[3,4]^ However, a stone in the SMG may not be found in many cases of Kuttner tumor. The salivary duct obstruction leads to excessive accumulation or retention of ductal secretions, which result in chronic inflammations.^[5,6]^ Immunoglobulin G4-related disease (IgG4-RD) is a recently recognized inflammatory disease in multiple organ systems.^[7]^ The salivary glands, particularly in the SMG, are frequently involved in the head and neck region. Nowadays, Kuttner tumor is known to be a chronic sclerosing sialadenitis, representing the focal manifestation of IgG4-RD.^[7]^

The clinical features of Kuttner tumor have diverse signs and symptoms, and diagnostic challenges significantly remained underrecognized and underscored for IgG4-RD. Kuttner tumor presents as a firm and painless enlargement of the SMG, and patients are advised to have surgical resection of the involved glandular tissue because of suspicion of possible benign or malignant tumors.^[7–9]^ The differential diagnoses include sialadenitis, Mikulicz syndrome, benign lymphoepithelial lesion, Kimura disease, lymphoma, and neoplasms of the salivary glands.^[10,11]^ For the proper diagnosis of Kuttner tumor of the SMG, surgeons mainly depend on preoperative ultrasonography with fine-needle aspiration cytology (FNAC) examinations. In the ultrasonogram, Kuttner tumor shows diffuse involvement with multiple hypoechoic lesions. Focal lesions are seen as hypoechoic, heterogeneous masses with a radial-branching vascular pattern within. The FNAC findings shows cells greatly reduced in number along with scattered tubular ducts against a backdrop of lymphoplasmacytic infiltration and fibrous deposits.^[12]^ There may be a reduced but moderate number of cells and ducts enveloped in fibrous sheaths, as well as fibrous proliferation of the gland’s septa.^[12]^ However, FNAC findings may not be specific for Kuttner tumor, and ultrasonography may exhibit similar imaging findings as neoplasm of the SMG. So diagnosis should require adjunct consideration of both the ultrasonogram and the clinical presentation.^[13]^ We therefore evaluate the immunologic features of IgG4-related Kuttner tumor to aid in proper diagnosis.

## 2. Subjects and Methods

We retrospectively chose 13 patients who were confirmed as having Kuttner tumor by surgical biopsy between May 2012 and January 2019 at the Department of Otolaryngology-HNS. We obtained proper consent from the patients in keeping with the mandate of the Declaration of Helsinki. Institutional Review Board was not required as it was a retrospective review. We had selected only 13 cases diagnosed as Kuttner tumor by SMG biopsies in this study period because many cases presenting with swelling of SMGs were diagnosed by clinical findings without biopsies. The diagnosis was made on the basis of clinical data, imaging, and histopathologic findings. For histological analysis, 4-μm formalin-fixed, paraffin-embedded sections were prepared and stained with hematoxylin and eosin. For immunohistochemical study, anti-IgG rabbit polyclonal antibody was used to evaluate the molecule of IgG (A0423; Dako, Glostrup, Denmark). Further, anti-IgG4 mouse monoclonal antibody was used to evaluate the molecule of IgG4 (MC011; Binding Site, Birmingham, UK). Marked plasmacyte infiltration, defined as >10 IgG4+ cells per high-power field (HPF) and a >40% ratio of IgG4+/IgG+ cells were considered as positive findings. They did not complain of drying eyes or mouth, but felt some discomfort on palpation of the masses and also postprandial. Their past medical histories were insignificant. Physical examination showed palpable submandibular masses unilaterally or bilaterally. To measure the IgG serum subclass, IgG and IgG4 serum levels were evaluated with the consent of our patients. Other serological data about the autoantibodies (Sjögren-syndrome-related antigen A [SSA]/Ro-antibody [Ab] and SSA/La-Ab) were also evaluated. The patient’s data was investigated using Excel software.

## 3. Results

**Figure 1.**
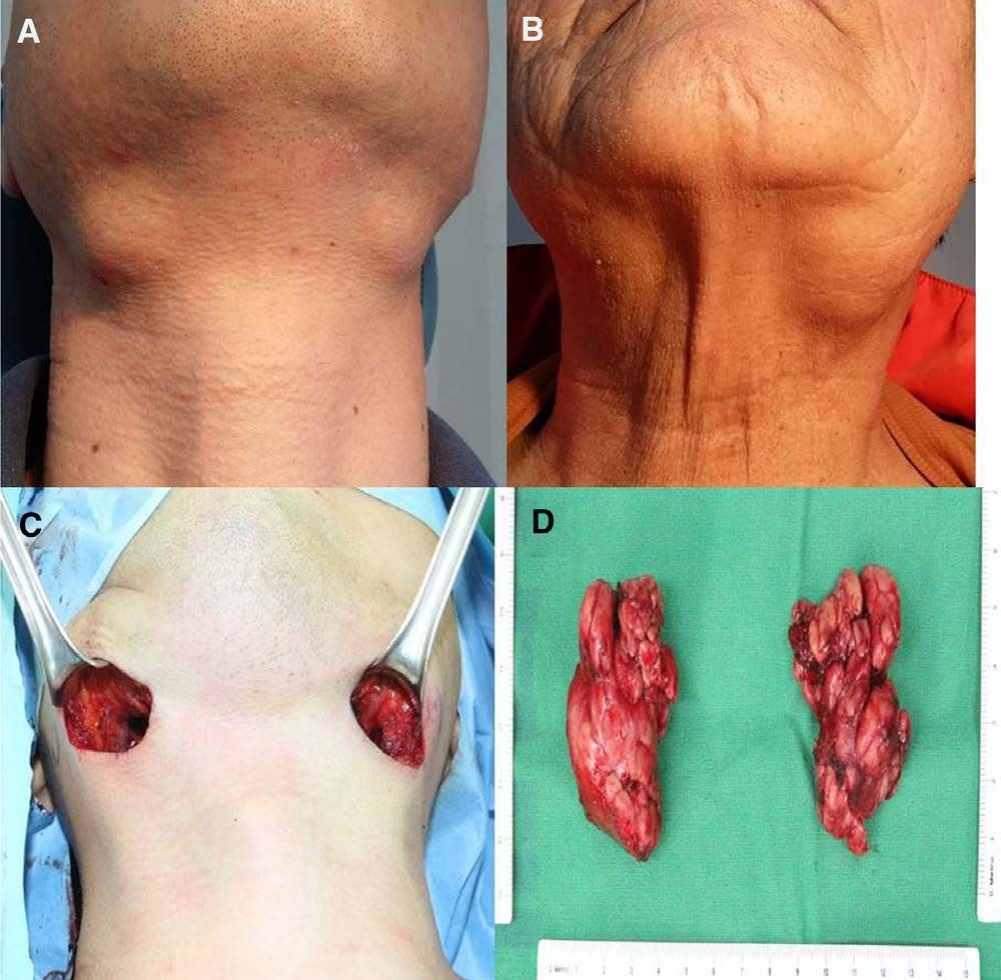
(A, B) Patients with bilateral mass in the submandibular glands. (C) Excision of bilateral submandibular glands, transcervically. (D) The excised specimens of Kuttner tumor.

The distributions of the patients are summarized in Table 1. Of the 13 cases included in this study, there were 9 male and 4 female patients. The age of the patients ranged from 41 to 83 years, with a mean age of 61.4 years. Seven patients received unilateral resection of SMG, and bilateral resection of SMG in 6 patients. Figure 1 shows the preoperative appearance of the patients and operative view.

**Table 1:** Clinicopathological features of the patients.

N	A/S	Site	FNA	Serum antibody		Serum IgG level		Pathology	
				SSA/Ro-Ab (U/mL)	SSA/La-Ab (U/mL)	IgG (mg/dL)	IgG4 (ng/mL)	IgG	IgG4
1	F/46	Both	Ch SA	2.7	4	1235.3	**2930**	(+)	(+)
2	F/48	Both		1.9	5.2	1548	**7760**	(+)	(+)
3	F/58	Both	LP	0.3	1.1	1459.7	**14,600**	(+)	(+)
4	M/47	Both	LP	0.8	1.4	1255	**10,900**	(+)	(+)
5	F/83	Left	Ch SA	2	3.4	**1750.3**	**9869.5**	(+)	(+)
6	M/74	Left	Ch SA	0.7	1.2	1212.9	**12,000**	(+)	(+)
7	M/75	Right		2.3	2.3	1216	**40,400**	(+)	(+)
8	M/63	Left	Ch SA	2	6.7	**5233.3**	**66,500**	(+)	(+)
9	M/63	Both	Ch SA	1.2	1.7	**1913.8**	**23,400**	(+)	(+)
10	M/65	Left						(+)	(+)
11	M/58	Right	Ch SA	1.6	2.5	1395	1650	(+)	(+)
12	M/64	Both	Ch SA	0.1	2.1	**1666.3**	**4010**	(+)	(+)
13	M/54	Right	LP	0.4	0.01	1563.5	**7710**	(+)	(+)

Normal levels: anti-SSA/Ro: 15–25, anti-SSA/La: 15–25, IgG: 700–1600, IgG4: 30–2010.

Ab = antibody, Ch SA = chronic sialoadenitis, F = female, FNA = fine-needle aspiration cytology, IgG = immunoglobulin G, LP = lymphoproliferative lesion, M = male, SSA = Sjögren-syndrome-related antigen A.

The following variables were assessed: FNAC findings, serum autoantibody levels (anti-Ro antibodies, anti-La antibodies), IgG serum levels (total IgG and IgG4), and immunohistochemical findings for IgG and IgG4 plasma cells.

For the FNAC studies, we had 10 cases of FNAC among the 13 cases. Of the 10 cases, 7 were reported as chronic sialoadenitis, and 3 as benign lymphoproliferative lesion. Unfortunately, there was no case suspicious of chronic sclerosing sialoadenitis or SMG tumors, perhaps because fine-needle aspiration was used, not core-needle biopsy.

**Figure 2.**
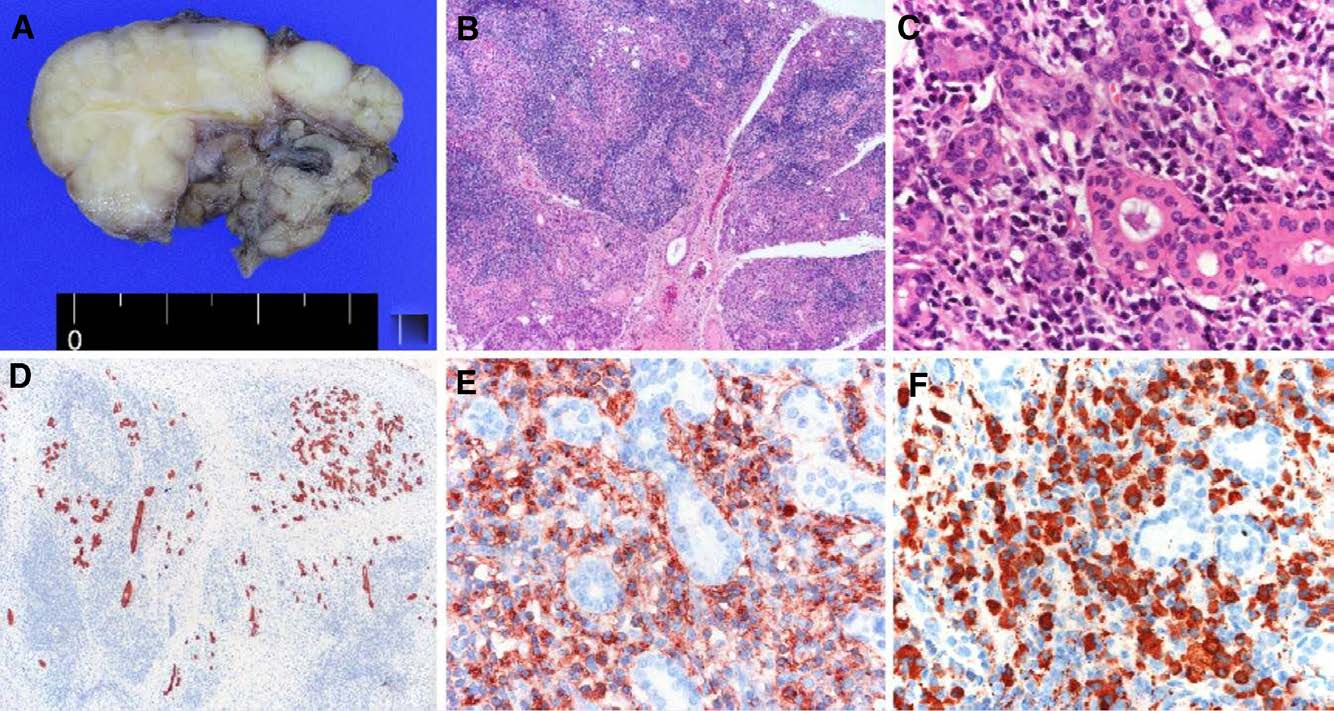
(A) The cut surface of the specimen. (B) Low-power view reveals lobules separated by fibrotic tissue. (C) High-power view reveals heavy infiltrates of lymphocytes and plasma cells. Immunohistochemical staining of cytokeratin (D), immunoglobulin G (E), and immunoglobulin G4 (F).

Figure 2 shows the histopathological findings. On the histological results of all cases who underwent resection of the SMG, unilateral or bilateral, the mass revealed many lobules separated by fibrotic tissue. Most of the area of the SMG was replaced by reactive lymphoid follicles and revealed encased glandular ducts and heavy infiltrates of lymphocytes as well as plasma cells. The lesions revealed encasement of glandular ducts and atrophy or loss of acini. On the immunohistochemical stain for cytokeratin accentuates, the lesions denote atrophic glandular ducts in the dense lymphoplasmacytic background. Immunohistochemical stain for IgG revealed many immunoreactive cells around the glandular ducts. Immunohistochemical stain for IgG4 revealed more than 100 immunoreactive cells around the glandular ducts. Generally, the minimum number of IgG4-positive cells for making the diagnosis of IgG4-RD in most tissues is from 30 to 50 per HPF. All patients showed all positive results for IgG4-RD, as well as for elevated numbers of IgG and IgG4-positive plasma cells in the immunohistochemical findings.

**Figure 3.**
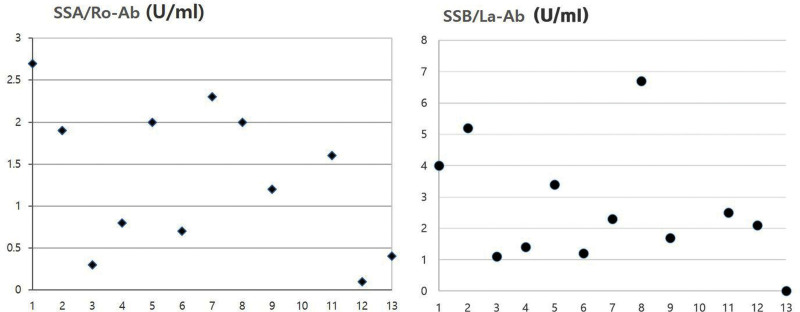
Distributions of SSA/R0-Ab and SSA/La-Ab levels. The level of autoantibodies are all normal ranges. Ab = antibody, SSA = Sjögren-syndrome-related antigen A.

Figure 3 shows the distribution of SSA/R0-Ab and SSA/La-Ab serum levels of the patients. On the serum level of autoantibodies (normal range; 15–25 U/mL), SSA/Ro-Ab showed all normal values of serum level, ranging from 0.1 to 2.6. SSA/La-Ab (normal range 15–25 U/mL) also showed all normal values of serum level, which ranged from 1.1 to 6.7.

**Figure 4.**
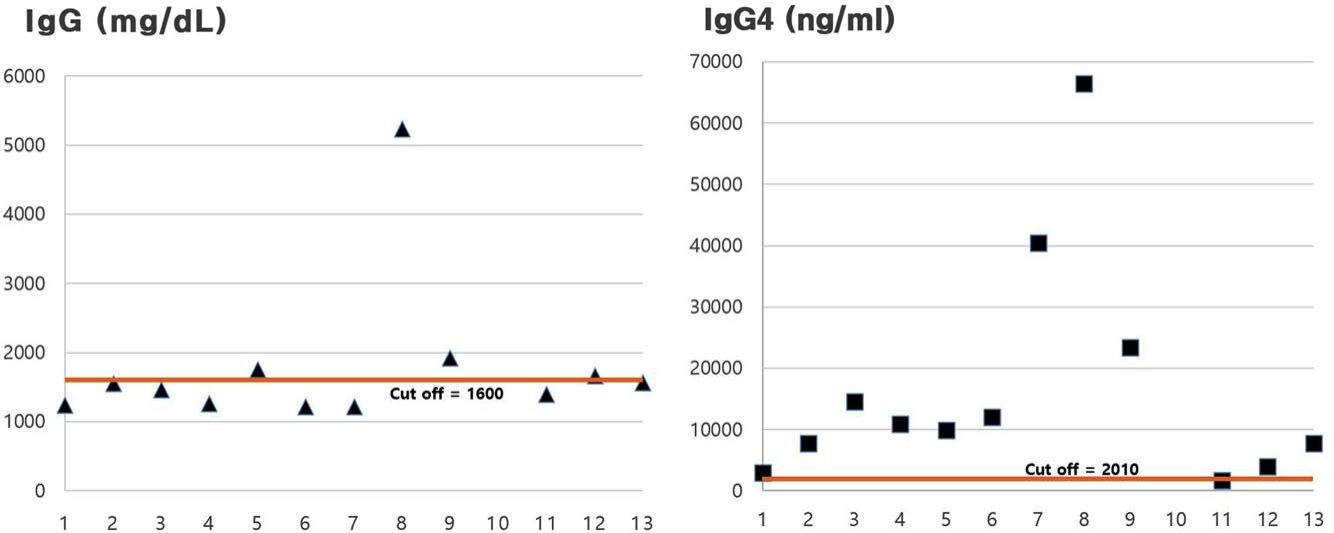
Distributions of IgG and IgG4 serum levels. The IgG serum levels are higher than normal in 3 cases, as IgG4 serum level is in 9 cases. The red lines stand for normal levels. IgG = immunoglobulin.

Figure 4 shows the distribution of IgG and IgG4 serum levels of the patients. On the serum level of IgG (normal range 700–1600 mg/dL) and IgG4 (normal range 30–2010 ng/mL), the serum level of IgG was increased in only 4 among the 12 cases, and 8 had normal levels. The IgG4 serum levels were significantly increased in 11 of 12 cases, but 1 case had a normal level.

## 4. Discussion

Recently, Kuttner tumor is known to be chronic sclerosing sialadenitis, representing the focal manifestation of IgG4-RD especially in the salivary glands. It can occur as part of systemic or isolated diseases.^[8]^ Systemic IgG4-RD more commonly occurs in older males, in a 1:0.77 male-to-female ratio. But there is no gender predilection; that is, there is equal male and female prevalence for IgG4 involvement in the head and neck.^[14]^

On the diagnostic procedures, FNAC analysis under ultrasound guidance is sometimes useful to confirm the diagnosis without recourse to organ excision, particularly when there is involvement of the SMGs. However, the patients are advised to have surgical resection of the SMG because of suspicion of possible benign or malignant tumors. It is only from postsurgical histopathology of the excised mass that the diagnosis of Kuttner tumor is definitively made. The key histopathological feature is the proportion of IgG4-positive plasma cells in the involved tissue, which could be diagnostic for excluding malignancy, especially including lymphoma.^[15]^ MALT lymphoma has similar imaging findings and always requires exclusion, because it can also occur secondarily to IgG4-RD within the salivary glands.^[16]^ Wah and John^[12]^ reported several features of FNAC for Kuttner tumor: relatively low cellularity, probably attributable to the fibrosis; scattered ductal structures with paucity or absence of acini; ducts intimately surrounded by collagen sheaths or lymphoid cells; small, isolated fragments of fibrous stroma; and moderate to large numbers of lymphoid cells that lack definite atypia. However, Chai et al^[13]^ have reported similar FNAC findings in chronic sialadenitis, broadly similar to those described for chronic sialadenitis or Kuttner tumor. Furthermore, recent study showed no correlation of IgG4+ plasma cells in histopathology sections with FNAC smears.^[17]^ Our FNAC results are benign lymphoproliferative lesion or reactive lymphadenitis, but are not diagnostic for the IgG4-RD within the SMG.

IgG is the most abundant type of antibody (approximately 80% of the total) and is found in all body fluids.^[18,19]^ It protects against bacterial and viral infections. The subclasses of immunoglobulin differ in their biological features and target specificity and are clinically interesting for potential immunodeficiencies and IgG4-RDs.^[18,19]^ Elevation of serum IgG4 was previously thought to be a key diagnostic finding, but patients with systemic disease may have normal serum levels, up to 50%. Furthermore, although relatively sensitive, the specificity of elevated IgG4 serum levels is low and not significant in many other, disparate diseases.^[20]^

Testing of IgG subclass levels may be indicated in patients with clinical evidence of a possible immunodeficiency or normal concentrations of total serum IgG. The IgG4 assay is best for IgG4-RD testing. Clinically, the causes of a reduced IgG serum level include acquired immunodeficiency, hereditary immunodeficiency, protein-losing syndromes, Waldenstrom macroglobulinemia, and non-IgG myeloma.^[14]^ The causes of a raised IgG include IgG myeloma, chronic liver disease, sarcoidosis, autoimmune disease, and parasitic diseases.^[14,18]^

Although the causes of autoantibody production are not well understood, it has been suggested that some autoantibodies are caused by a genetic predisposition combined with an environmental trigger, such as a virus or a prolonged exposure to certain toxic chemicals. Autoantibodies directed against Ro/SSA and La/SSB autoantigens were originally identified in patients with Sjögren syndrome and systemic lupus erythematosus (SLE).^[19]^ Subsequent studies showed that anti-Ro/SSA antibodies may be present in patients with other autoimmune diseases, including systemic sclerosis, idiopathic inflammatory myopathies, primary biliary cholangitis, and rheumatoid arthritis. In contrast to anti-Ro antibodies, which may be present in a variety of autoimmune diseases, anti-La antibodies are specific for the diagnosis of SLE and Sjögren syndrome.^[20]^ In addition, as with anti-Ro antibodies, anti-La antibodies may be detected in the mothers of children who are born with neonatal lupus syndrome. The combination of anti-Ro and anti-La antibodies is relatively specific for the diagnoses of SLE and Sjögren syndrome.^[18]^

Routine neck computed tomography and magnetic resonance imaging (MRI) show an enlarged focal mass or diffuse infiltrating lesions. However, computed tomography and MRI are also less specific, and there is subtle enlargement of the involved gland, relative to the adjacent normal parenchyma. MRI may reveal a relatively hypointense mass on T2-weighted sequences that is indistinguishable from lymphoma.^[21]^

Unfortunately, for the confirmed diagnosis of Kuttner tumor, open biopsy or excision should be performed to rule out other benign lesions or malignancies, including a lymphoma. For the histological features, it needs to present at least 2 of 3 histological features, such as dense lymphoplasmacytic infiltrate, storiform fibrosis, and obliterative phlebitis. In most cases, the diagnosis is reached with evidence of the lymphoplasmacytic infiltrate and fibrosis. Immunohistochemical staining needs to present elevated numbers of IgG4-positive plasma cells within tissue in Kuttner tumor related to IgG4-RD.^[9,13]^ The number of IgG4-positive cells per HPF is counted and averaged over 3 fields; in general, more than 50 per HPF is required. In addition, the proportion of IgG4-positive to total IgG-positive plasma cells is estimated; in general, a ratio of 40% is accepted as diagnostic.^[18]^ In our cases, all patients were confirmed as having Kuttner tumor by showing elevated numbers of IgG4-positive plasma cells in the involved tissue. Furthermore, the IgG4 serum levels were significantly increased in 11 of 12 cases. However, elevated serum IgG4 concentrations could also be found in patients with atopic dermatitis, pemphigus, asthma, and multicentric Castleman disease.^[22]^ This study has a limitation in relatively small sample size with and retrospective study design. Therefore, prospective study with large sample size is required in the near future.

## 5. Conclusion

IgG4-related Kuttner tumor in the SMG is a recently recognized inflammatory disease with typical immunohistopathological findings. Imaging studies may exhibit similar findings to those of other benign lesions in the SMG, and FNAC findings may not be specific for Kuttner tumor. Immunological study, including serum IgG and IgG4 levels, should be required for proper diagnosis with the clinical features of the Kuttner tumor. Especially, this study might be very informative for patients with Kuttner tumor, which has mild symptom avoiding improper surgical resection of the SMG. Further, this study expected to provide more information about Kuttner tumor to physicians and researchers.

## Author contributions

**Supervision:** Yong Tae Hong.

**Writing – original draft:** Su Geun Kim.

**Writing – review & editing:** Yong Tae Hong, Chan Mi Lee.

## References

[1] Chan JKC. Kuttner tumor (chronic sclerosing sialadenitis) of the submandibular gland: an under-recognized entity. Adv Anat Pathol. 1998;5:239–51. 9859756 10.1097/00125480-199807000-00004

[2] Rasanen O Jokinen K Dammert K. Sclerosing inflammation of the submandibular salivary gland (Kuttner tumor). A progressive plasmacellular ductis. Acta Otolaryngol. 1972;74:297–301. 5076598 10.3109/00016487209128454

[3] Harrison JD Epivatianos A Bhatia SN. Role of microliths in the aetiology of chronic submandibular sialadenitis: a clinicopathological investigation of 154 cases. Histopathology. 1997;31:237–51. 9354894 10.1046/j.1365-2559.1997.2530856.x

[4] Huang C Damrose E Bhuta S. Kuttner tumor (chronic sclerosing sialadenitis). Am J Otolaryngol. 2002;23:394–7. 12430136 10.1053/ajot.2002.126855

[5] Adachi M Fujita Y Murata T. A case of Kuttner tumor of the submandibular gland. Auris Nasus Larynx. 2004;31:309–12. 15364370 10.1016/j.anl.2004.05.008

[6] Williams HK Connor R Edmondson H. Chronic sclerosing sialadenitis of the submandibular and parotid glands: a report of a case and review of the literature. Oral Surg Oral Med Oral Pathol Oral Radiol Endod. 2000;89:720–3. 10846127 10.1067/moe.2000.102515

[7] Stone JH Zen Y Deshpande V. IgG4-related disease. N Engl J Med. 2012;366:539–51. 22316447 10.1056/NEJMra1104650

[8] Hong X Sun ZP Li W. Comorbid diseases of IgG4-related sialadenitis in the head and neck region. Laryngoscope. 2015;125:2113–8. 25994602 10.1002/lary.25387

[9] Li W Chen Y Sun ZP. Clinicopathological characteristics of immunoglobulin G4-related sialadenitis. Arthritis Res Ther. 2015;17:186. 26194097 10.1186/s13075-015-0698-y PMC4508811

[10] Chan JK. Kuttner tumor (chronic sclerosing sialadenitis) of the submandibular gland: an underrecognized entity. Adv Anat Pathol. 1998;5:239–51. 9859756 10.1097/00125480-199807000-00004

[11] Kojima M Nakamura S Itoh H. Sclerosing variant of follicular lymphoma arising from submandibular glands and resembling Kuttner tumor: a report of 3 patients. Int J Surg Pathol. 2003;11:303–7. 14615825 10.1177/106689690301100407

[12] Wah C John KCC. Kuttner tumor of the submandibular gland fine-needle aspiration cytologic findings of seven cases. Anat Pathol. 2002;117:103–8. 10.1309/G9T3-22MH-Q7KL-G2DL 11791589

[13] Chai C Dodd LG Glasgow BJ. Salivary gland lesions with a prominent lymphoid component: cytologic findings and differential diagnosis by fine-needle aspiration biopsy. Diagn Cytopathol. 1997;17:183–90. 9285189 10.1002/(sici)1097-0339(199709)17:3<183::aid-dc3>3.0.co;2-g

[14] Kitagawa S Zen Y Harada K. Abundant IgG4-positive plasma cell infiltration characterizes chronic sclerosing sialadenitis (Kuttner’s tumor). Am J Surg Pathol. 2005;29:783–91. 15897744 10.1097/01.pas.0000164031.59940.fc

[15] Kojima M Miyawaki S Takada S. Lymphoplasmacytic infiltrate of regional lymph nodes in Kuttner’s tumor (chronic sclerosing sialadenitis): a report of 3 cases. Int J Surg Pathol. 2008;16:263–8. 18573783 10.1177/1066896907306969

[16] Ochoa ER Harris NL Pilch BZ. Marginal zone B-cell lymphoma of the salivary gland arising in chronic sclerosing sialadenitis (Kuttner tumor). Am J Surg Pathol. 2001;25:1546–50. 11717546 10.1097/00000478-200112000-00012

[17] Kaur R Mitra S Rajwanshi A. Fine needle aspiration cytology of IgG4 -related disease: a potential diagnostic pitfall? Diagn Cytopathol. 2017;45:14–21. 27666423 10.1002/dc.23617

[18] Culver EL Bateman AC. General principles of IgG4-related disease. Diagnostic Histopathol. 2013;19:111–8.

[19] Kamisawa T Zen Y Pillai S. IgG4-related disease. Lancet. 2015;385:1460–71. 25481618 10.1016/S0140-6736(14)60720-0

[20] Ferry JA Deshpande V. IgG4-related disease in the head and neck. YSDIA. 2012;29:235–44. 10.1053/j.semdp.2012.07.008 23068303

[21] Ruberto E Gangemi E Covello R. MRI features in submandibular gland chronic sclerosing sialadenitis: a report of three cases and imaging findings. Iran J Otorhinolaryngol. 2020;32:397–401. 33282789 10.22038/ijorl.2020.47418.2583 PMC7701483

[22] Umehara H Okazaki K Masaki Y. Comprehensive diagnostic criteria for IgG4-related disease (IgG4-RD), 2011. Mod Rheumatol. 2012;22:21–30. 22218969 10.1007/s10165-011-0571-z

